# A comprehensive analysis of multi-circulatory disorders in early pressure injury and their diagnostic significance in rat models

**DOI:** 10.1038/s41598-023-46676-x

**Published:** 2023-11-07

**Authors:** Lu Chen, En Takashi, Akio Kamijo, Daiji Miura, Jian Lu, Lan Zhang, Hirotomo Ten, Jianglin Fan

**Affiliations:** 1 Division of Basic & Clinical Medicine, Faculty of Nursing, Nagano College of Nursing, Komagane, Japan; 2 Department of Molecular Pathology, Graduate School of Medical Sciences, Faculty of Medicine, University of Yamanashi, Yamanashi, Japan; 3 Device Technology Research Institute, National Institute of Advanced Industrial Science and Technology, Tsukuba, Japan; 4 Department of Judo Physical Therapy, Faculty of Health, Teikyo Heisei University, Tokyo, Japan

**Keywords:** Cardiology, Health care, Pathogenesis

## Abstract

Early pressure injury (PI) progression is associated with multi-circulatory disorders and they interplay with each other, resulting in a lack of a satisfactory diagnostic method. We generated early PI and blanchable erythema hairless rat models. Transparent disc method and capillary refilling time test (CRTT) results were recorded with ultraviolet camera to capture the dynamics changes, and the blanching index and refilling index were set for comprehensive analysis. The deteriorated areas of early PI showed non-blanchable erythema (NBE) and an increase in erythema at 0.5 and 6 h with the transparent disc method. CRTT showed a marked refilling delay at 12 h. The comprehensive analysis of blanching index and refilling index showed a significant change in erythema from NBE at 0.5 h and ischemia progressing to hemorrhage at 18 h. There was also a marked difference in the deteriorating and improving areas within the same erythema. Pathological analysis showed inflammatory cell infiltration, with marked edema accompanied by increased hemorrhage and tissue necrosis. Furthermore, small arteries and veins with thrombosis and microthrombi were observed. Consistent ischemia after decompression and subsequent hemorrhage are important indicators, and comprehensive analysis can help increase the positive diagnosis rate over that for other circulatory disorders alone.

## Introduction

Pressure injury (PI), which is localized damage to the skin and underlying soft tissue, has become a serious problem affecting patients’ quality of life and increasing the financial burdens^1^. Therefore, the early diagnosis and treatment to prevent the further progression of PI are very important.

According to the guideline of the National Pressure Injury Advisory Panel (NPIAP), the earliest stage 1 (Early PI) is defined as intact skin with a localized area of non-blanchable erythema (NBE) measured by the transparent disc method caused by intradermal hemorrhage and can be distinguished from the blanchable erythema (BE), which is caused by hyperemia^1^. Transparent disc method adopts transparent plate or finger pressure erythema to see its fading condition. Although it is simple and feasible, it cannot fully reflect the changes of early PI and has a high misdiagnosis rate. The transparent disc method is simple and easy to perform, but can not fully reflect the changes of early PI and has a high rate of misdiagnosis.

Early PI is known to progress from erythema to deterioration into ulceration after decompression. It can take up to 48 h from the onset of the injury to hemorrhage by macroscopic detection^2^. However, few studies have focused on changes in early PI before hemorrhage. In addition, numerous studies have pointed out that visual skin examinations are unreliable, as there is a high probability of severe skin damage before identification^3,4^. To gather more information, Scafide et al.^5^ suggested using assessment tools to improve the ability to diagnose early PI.

More importantly, many reports have suggested that early PI is not simply a matter of hemorrhage, such as the presence of thrombosis^6,7^, which leads to the ischemia or congestion is rarely reported and the impact of these lesions on early PI is even less discussed. In addition, reperfusion and hyperemia caused by inflammation are also involved in the progress of PI. To identify these complex circulatory disorders, we applied the principle of ultraviolet (UV) absorption (405 nm) by hemoglobin (Hb) and found that UV photography could detect hyperemia, congestion and hemorrhage in early PI earlier and more accurately than macroscopic observation^8^. For the evaluation of ischemia, the capillary refill time test (CRTT) can be used to detect the dynamics of peripheral circulation. We confirmed the presence of ischemia using biomarkers and a spectrophotometer to record the presence of ischemia based on the CRTT in early PI^9^. However, which of these circulatory disorders is more relevant for the diagnosis during the deterioration of early PI remains unclear.

In the present study, we constructed animal models of early PI, with blanchable erythema (BE) as a control group. To identify more meaningful indicators for distinguishing the two from the complex pathology, our hypothesis is to utilized UV photography and combined both the transparent disc method and CRTT to evaluate the dynamics of various circulatory disturbances after decompression and thereby develop a more useful method for detecting early PI.

## Materials and methods

### Animals

Five 8-week-old male hairless rats (HWY/Slc; SLC, Inc. Shizuoka, Japan) were used in this experiment. The animals were maintained on a standard chow diet with ad libitum access to water and in a 12-h light/dark cycle throughout the experiment. All experiments were conducted in accordance with the recommendations in the Guide for the Care and Use of Laboratory Animals of the National Institutes of Health. The protocol was approved by the Committee on the Ethics of Animal Experiments of the Nagano College of Nursing (protocol code: No. 2021-5; date of approval: November 1, 2021).

### Generation of BE and early PI models

**Figure 1:**
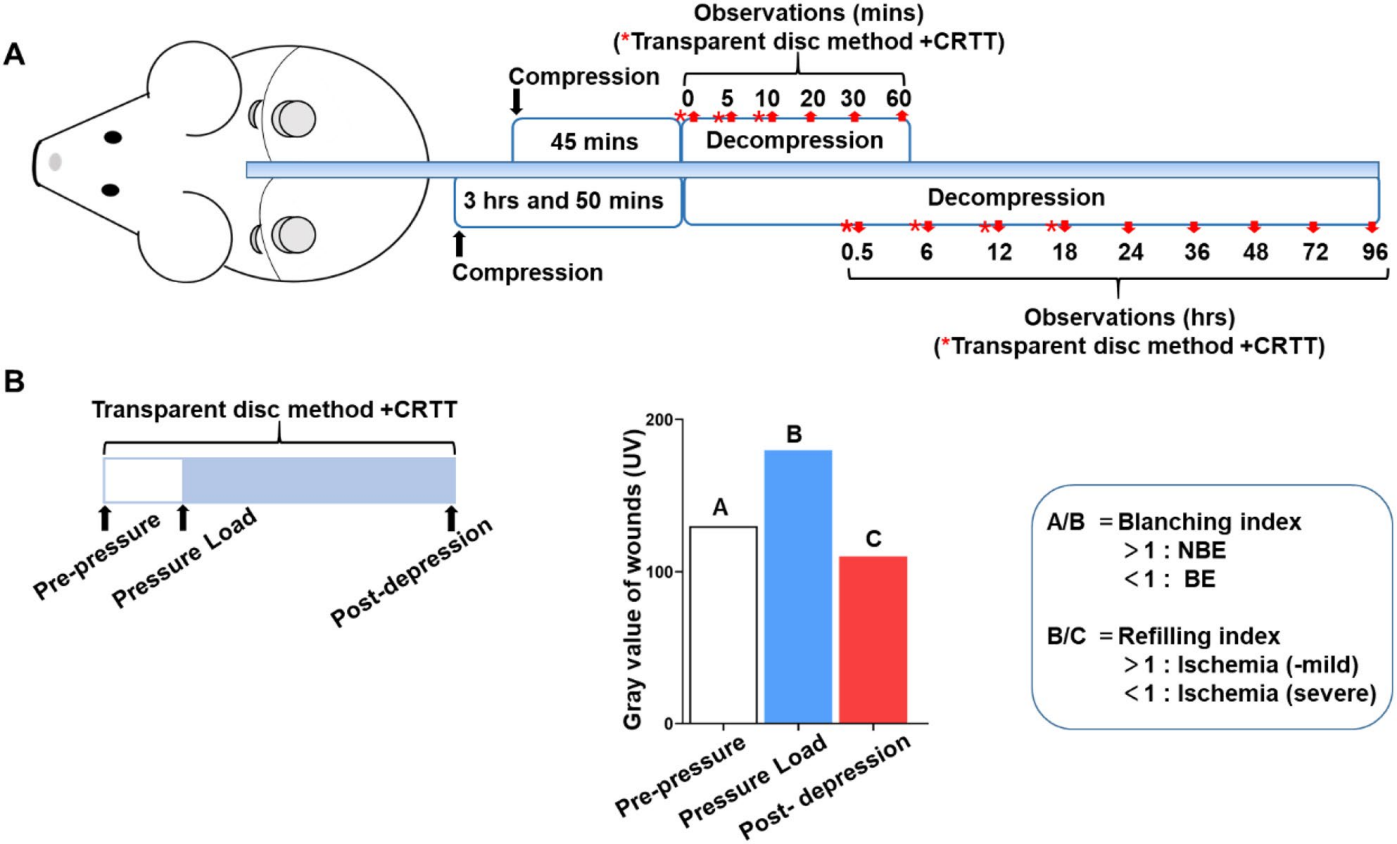
(**A**) Schematic illustration of the construction of models. The models were established by pressing the dorsal skin for different times between two circular neodymium magnets (10 mm in diameter). The procedure for detecting ischemia at different times after decompression using CRTT coupled with UV photography methods. *Transparent disc method + CRTT. (**B**) CRTT was performed coupled with the transparent disc method by applying temporary pressure at 150 mmHg in the BE and early PI groups (upper). A represent the gray values at pre-pressure, B indicates the gray value at pressure load, and C indicates the gray value post-depression (bottom). BE, blanchable erythema.

Two rat models representing either BE or early PI were made and analyzed according to the methods of a previous study^9^. In brief, hairless rats were anesthetized with isoflurane inhalation (import: 4–5%; maintenance: 2–3%) (Meiji Seika Pharma Co., Ltd. Tokyo, Japan). For the generation of the BE model, symmetrical part of the left or right back skin was gently pulled up and then pressed between two circular neodymium magnets (10 mm in diameter × 4 mm in thickness) (Niroku Seisakusho Co., Ltd, Japan) with 440 mmHg of attraction. In the BE group, the press time was 45 min. In the early PI group, the press time for 3 h and 50 min (Fig. 1).

### Macroscopic observations

Photographs of the wounds were taken with a digital camera (Optio WG-3 PENTAX, Japan) at the start, and 5, 10, 20, 30 and 60 min after decompression in the BE group and 0.5, 6, 12, 18, 24, 36, 48, 72, and 96 h after decompression in the early PI group. The distance between the skin wounds and the camera was fixed at 20 cm. The optical magnification was set to 4×.

### Evaluating of ischemia using a dermal camera

#### Dermal camera photography

A Dermo-camera (DZ-D100, CASIO Co. Japan) was used to record photos of the PI wounds. DERMO CONT mode was selected as moderate light intensity. The lens of the dermal camera close adherence to on the wounds for continuous photography with white and UV LED light. We also captured photos of the wounds at the start, and 5 and 10 min after decompression in the BE group and at 0.5, 6, 12, 18, 24 and 36 h after decompression in the early PI group.

#### Gray value measurement of wounds

The Image Analysis Software (Image J, NIH) was used to analyze the UV photographs taken by the dermal camera. The gray values of the wounds area (10 mm in diameter) in the 2 groups were calculated. Typically, the gray values range from 0 (black) to 255 (white). In this study, the gray value reflected the ischemia situation of erythema.

**Figure 2:**
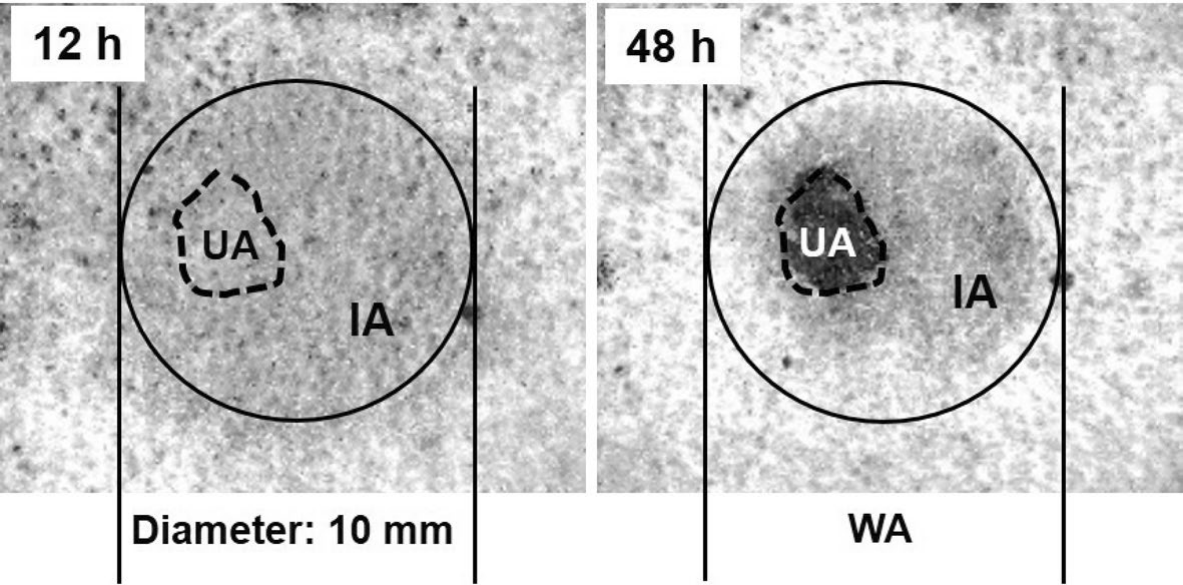
Ultraviolet photographs of the pressure injury (PI) wound and gray values of each area in the early PI group. The left shows the PI wound at 12 h after decompression, and the right shows the same area at 48 h when the ulcer had developed. WA, whole area; UA, ulcer area; IA, improvement area.

The early PI group is based on 48 h and the area where the ulcer appeared represented the area of deterioration (UA), the area around the ulcer represented the improvement area (IA), and the sum of the two was the whole area of pressure (WA) (Fig. 2).

### Measurement of ischemia—the transparent disc method and CRTT

To simulate the transparent disc method and CRTT, we fixed the Dermo-camera on a digital force gauge (DST series; Imada Co., Toyohashi, Japan). The wound area of the rats was placed on a round platform (2 cm in diameter) and then compressed. As described previously^9^, the pressure applied in the current study was set at 150 mmHg. The pressure instrument used an electric stand (MX500N; Imada Co., Toyohashi, Japan) to regulate the pressure. The rats were anesthetized to maintain sedation and thus ensure the accuracy of the applied pressure. For CRTT, we capture pictures with the Dermo-camera for three time periods perfusion observation: before (pre-pressure), at the application of the pressure load and 3 s after depression (post- depression).

We started to observe changes in the erythema at the start, and at 5, and 10 min post-decompression in the BE group. For the early PI group, we observed changes in the erythema at 0.5, 6, 12, and 18 h post-decompression.

To perform the CRTT, we started to observe changes in the erythema at the start, and at 5, and 10 min post-decompression in the BE group. For the early PI group, we observed changes in erythema at 0.5, 6, 12, 18 h post-decompression. We started to observe the changes in erythema at the start, 5, 10 min during post-decompression in the BE group. For the early PI group, we observed the changes in erythema at 0.5, 6, 12, 18 h during post-decompression.

### Boundary values of the diagnosis

For ease of observation, based on the results of the transparent disc method and CRTT, the gray values of UV were set as follows: the pre-pressure value was set as A, the value for a pressure load of 150 mmHg was set as B, and the value after decompression was set as C. The pre-pressure/pressure load (A/B) represents the blanching index, showing the situation of the transparent disc method, while pressure load/post-depression (B/C) represents the refilling index, which shows the increased refilling volume (Fig. 1B).

The blanching index and refilling index were analyzed together, and the ratio of each sample was resolved using a proportional function Y = X to set the boundary value.

In addition, according to the setting standard, we analyzed and compared the ratios of values of each sample, and calculated the positive diagnosis rate according to the diagnostic criteria in the two groups.

### Pathological analysis

The other four rats were used for pathological observation, and the models werethe same as above. Then we intravenously injected black ink solution which was considered to distinguish vascular hyperemia or clots from thrombi as previously reported^9^. The skin specimens were collected at 24 h after decompression and fixed in a 10% neutral formalin solution. All specimens were embedded in paraffin and cut into sections (5 μm thickness). Sections were then stained with hematoxylin and eosin (HE). Histological features were observed under a light microscope to evaluate the arteriovenous thrombosis, inflammatory changes and tissue necrosis. The skin specimen was also stained with Masson’s trichrome stain to confirm scattered red blood cells (RBCs) in tissue to confirm hemorrhage and microthrombi.

### Statistical analysis

All values are expressed as the means ± SEM. Statistical tests were conducted using the GraphPad Prism 7.0 software program (GraphPad Software, San Diego, CA, USA).

The collected data were entered into a database with the Excel 2016 software (Microsoft Office 2016). Data were examined by the Shapiro–Wilk test to verify their distribution. An unpaired-sample t-test was used to analyze parametric distributed data. In all cases, a *p* value of less than 0.05 was considered statistically significant.

### Ethical approval

The study is reported in accordance with ARRIVE guidelines.

## Results

### Macroscopic observations

**Figure 3:**
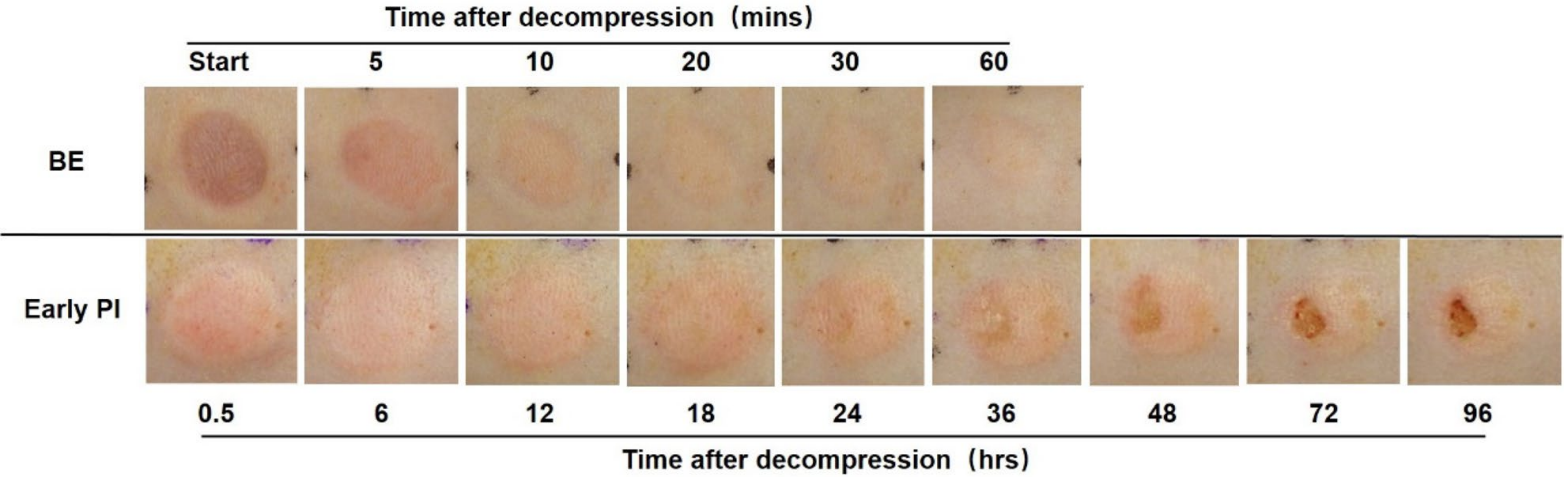
The representative images of macroscopic observation of the two models. In the BE group (upper), the erythema became reddish first but gradually faded until it almost disappeared at 60 min after decompression. In contrast, in the early PI group (bottom), the erythema showed a little bit pinkish and appeared the features of ulcer at 96 h after decompression. BE, blanchable erythema.

To observe changes in the two groups, we conducted long duration observations with a digital camera. BE lesions became obvious early on but were almost invisible (healed) after 60 min (upper) whereas early PI lesions remained remarkable until 24 h after decompression. During the period, slight increases or decreases were seen. The gross lesions of early PI apparently showed the features of ulcers at 48 h and formed a complete ulcer at 96 h (bottom) (Fig. 3).

### Dermal camera observation

**Figure 4:**
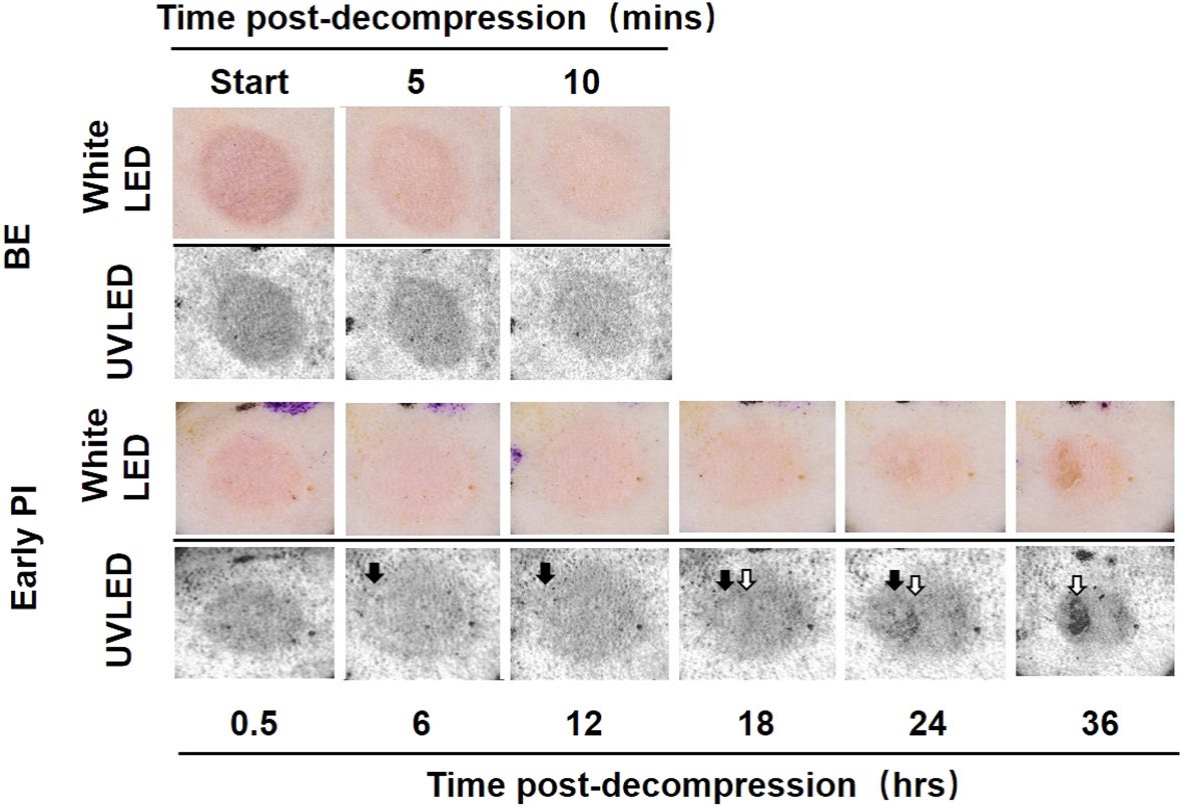
Photos taken by the dermal camera (visual and UV images) at different times after decompression in the two groups. The UV shadow showed even but decreased over time in BE group. In contrast, the UV shadow shows even at 0.5 h and pan-white area appeared at 6 h in the early PI group. BE, blanchable erythema.

The progress of the erythema in the two groups is shown in Fig. 4. The UV shadow in the BE group was visually uniform, and it gradually decreased with the passage of time. In contrast, in the early PI group, the UV shadow became relatively uniform at 0.5 h, and a whitish flushed area of inadequate blood flow occurred within the UV shadow at 6 h (black arrow). In addition, the thick and thin uneven shadow enhanced and enlarged in the pan-white area at 18 to 24 h after decompression (hemorrhage, white arrow). Thereafter, it deteriorated into an ulcer centered on the pan-white area within the erythema. These above-mentioned variations are not apparent with white-light LEDs.

### Visual observations with the transparent disc method and CRTT by white and UV LED

The CRTT results were observed at 5 min after decompression in the BE group (upper) and at 6 h before decompression as well as, during and after decompression in the early PI group (bottom).

**Figure 5:**
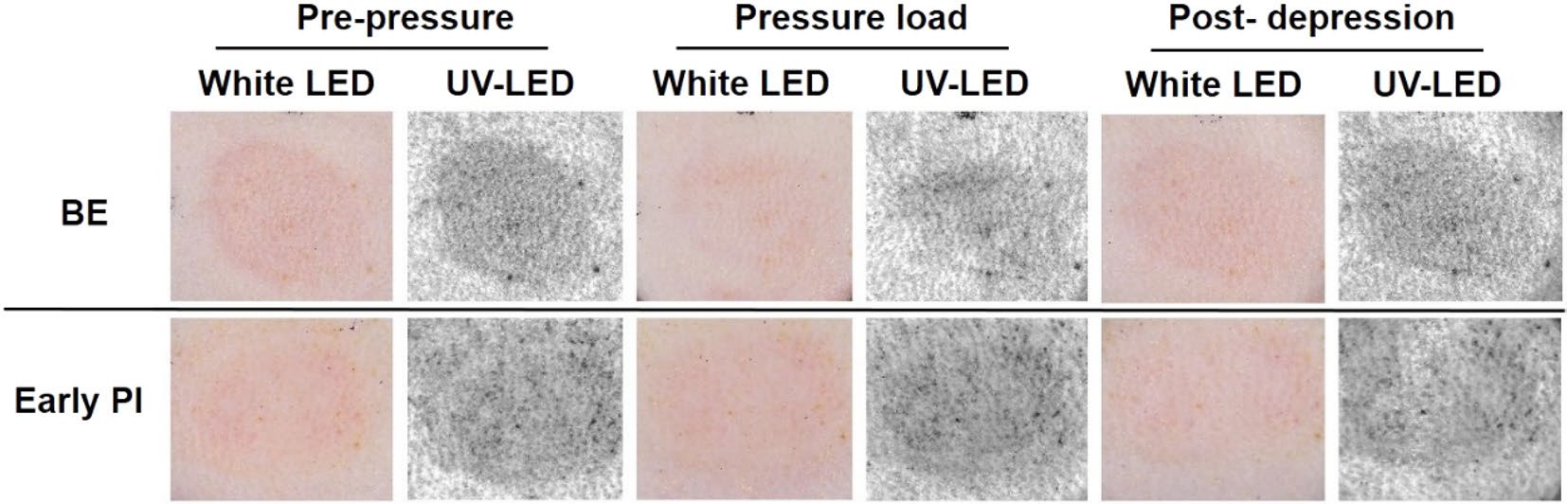
The visual observation of CRTT by white and UV LED. Photos taken by the dermal camera coupled with CRTT. The two groups were collected at the pre-pressure period, pressure load and post-depression. The UV shadow was significantly reduced after compression but rapidly recovered after CRTT. In the early PI group, pan-white area showed different trend with CRTT. BE, blanchable erythema.

The UV shadows were significantly reduced in the BE group after decompression, but there was still a mild residual. The UV shadow recovered rapidly after CRTT. In the early PI group, there was a partial whitish area in the UV shadow before pressure loading; however, the UV shadows were instead aggravated overall but the whitish area became unknown. In contrast, the CRTT, it showed an enlarged whitish area (Fig. 5).

### Gray values of UV shadows

**Figure 6:**
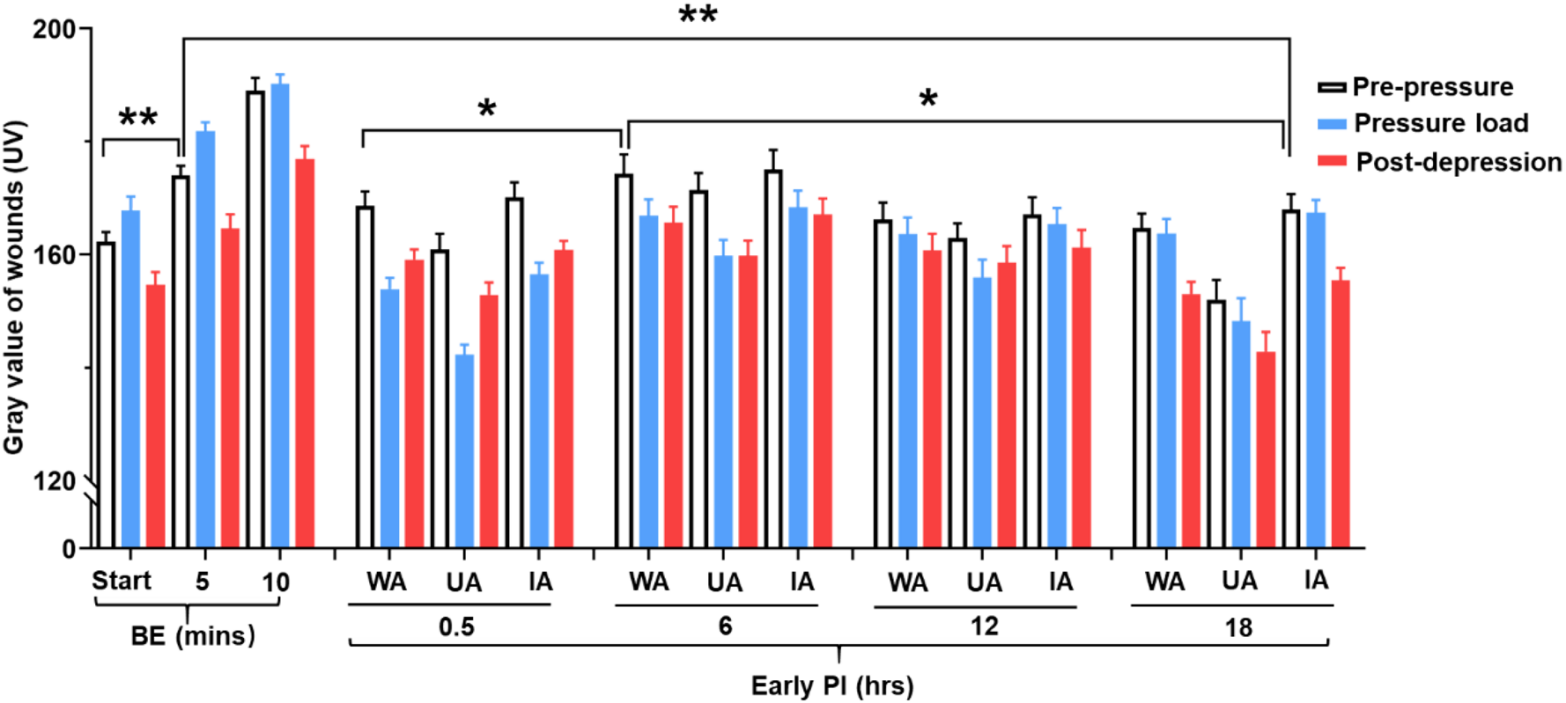
Quantitation of UV gray values by the transparent disc method and CRTT. Gray values at different times after decompression with transparent disc method and CRTT in the two groups. The UV gray value represents three points: pre-pressure, pressure load (150 mmHg) and after depression. In BE group, the results showed the gray values at the start, 5 min and 10 min after decompression using CRTT. In the early PI group, the results showed the gray values of WA, UA and IA at different times after decompression. WA, whole area; UA, ulcer area; IA, improvement area. n = 10 for each group. *< 0.05 **< 0.001. BE, blanchable erythema.

As shown in Fig. 6, the gray values of the UV images were measured with the Image J software. In the BE group, the UV shadow gray values were lowest, gradually increasing at 5 and 10 min, thereby, reflecting the weakening of the erythema. The gray values showed an increasing trend during the pre-pressure period and a decreasing trend on the CRTT at the three time points after decompression, a finding consistent with the macroscopy results (Fig. 5).

In the early PI group, the gray values of the WA, UA and IA showed a slight increase or decrease at pre-pressure. There was a significant increase in the gray value at 6 h compared to 18 h (*p* < 0.05), and while there was a decrease in all regions on application of the pressure load. There was also an increase or flattening of the gray values of UA at 0.5 to12 h but a slight decrease at 18 h. The gray values of WA and IA showed a decreasing trend, except for an increase that was seen at 0.5 h. (Supplemental data).

### Individual and comprehensive analysis of the blanching and refilling indexes

**Figure 7:**
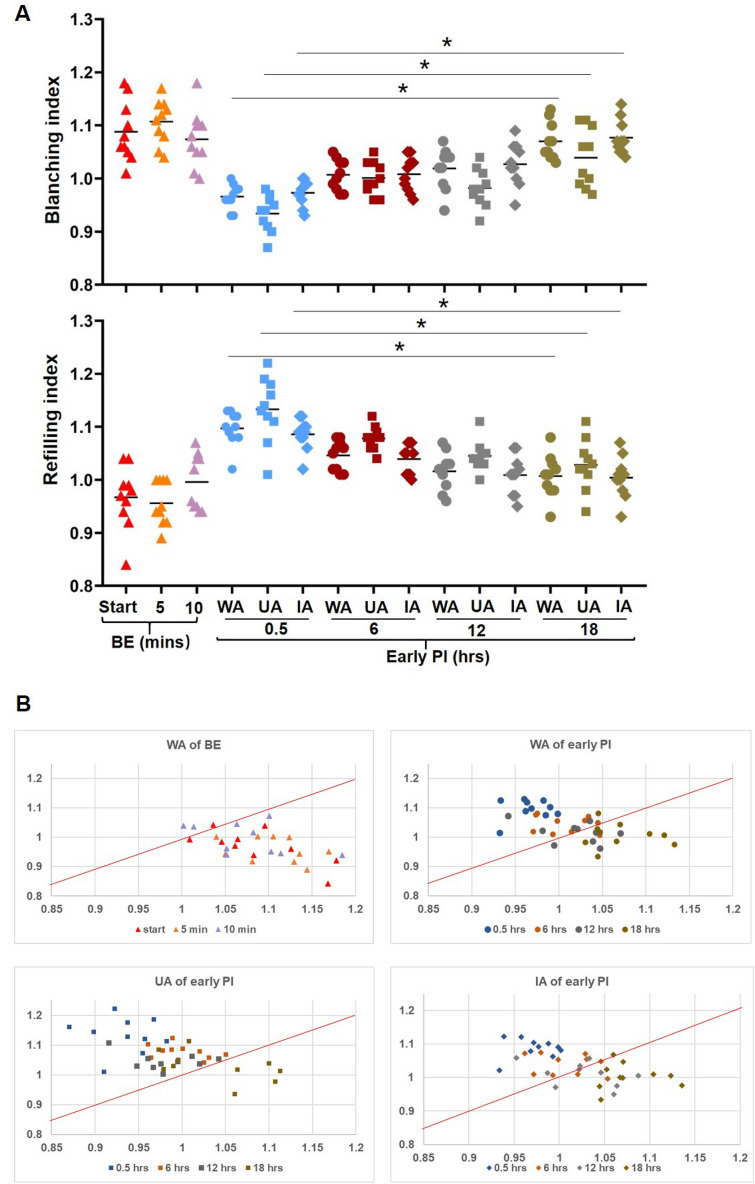
(**A**) The analysis of blanching index (upper) and refilling index (bottom). Gray values ratios at different times after decompression in the two groups. Blanching index (pre-pressure/pressure load) above 1 was considered as NBE while below 1 was considered as BE. Refilling index (pressure load/post-depression) above 1 was considered as mild ischemia while below 1 was considered as severe ischemia. Erythema in the early PI group increased from a delayed refilling at 0.5 h to a subsequent increase in refilling, especially at 18H in WA, UA and IA. n = 10 for each group (*p < 0.001 vs. 0.5 h). (**B**) Comprehensive analysis of blanching index and refilling index. Based on the distribution of values, Y = X (red dotted line) was set as a proportional to distinguish between the WA of BE group below the boundary and the WA, UA and IA of the early PI group above the boundary. WA, whole area; UA, ulcer area; IA, improvement area. Each point represents time after decompression. n = 10 for each group. BE, blanchable erythema.

The blanching index showed an increase in the erythema in the early PI group from non-blanchable at 0.5 h to subsequently fading, a finding that was more pronounced at 18 h in UA and IA within the erythema (*p* < 0.001 vs. 0.5 h). In addition, the UA and IA within the same erythema exhibited obvious different. In the BE group, the erythema showed blanching and with little variation over time (Fig. 7A, upper).

The refilling index showed that a delay refilling from 0.5 h to a subsequent increase , a finding that was more pronounced at 18 h in UA and IA within the erythema in the early PI group (*p* < 0.001 vs. 0.5 h). In addition, the UA and IA within the same erythema were markedly different. In the BE group, the erythema showed sufficient refilling with little variation over time (Fig. 7A, bottom).

Our comprehensive analysis showed the fading and refilling displacements of the two groups, giving a better indication of the differences and direction of progression of the two groups than when evaluated separately. Of note, setting Y = X as a proportional function clearly distinguished between the BE group below the boundary and the UA of the early PI group (above the boundary), while the WA and IA values fell between them (Fig. 7B).

### The diagnostic accuracy rate

Table 1 shows the result of the comprehensive analysis using the blanching and refilling indexes, with a proportional function to set the boundary values. The distributions in the BE group at the start and 5 min after decompression were set below the boundary values with an accuracy of 100% and 80% accuracy rate at 10 min. The ratio distribution of early PI group was set as accuracy above the boundary value, and the UA accuracy rate reached 100% from 0.5 to12 h, but dropped to 50% at 18 h. The WA and IA ratios were above and below the boundary value, respectively.

**Table 1:** The accuracy diagnostic rate of BE and early PI.

	Accuracy diagnosis rate				
	Time after decompression	0 min	5 min	10 min	
BE N = 10	WA	100% (0:10)	100% (0:10)	80% (2:8)	
Early PI N = 10	WA	100% (10:0)	90% (9:1)	50% (5:5)	10% (1:9)
	UA	100% (10:0)	100% (10:0)	100% (10:0)	50% (5:5)
	IA	100% (10:0)	80% (8:2)	50% (5:5)	10% (1:9)
	Time after decompression	0.5 h	6 h	12 h	18 h

n = 10 for each group.

WA, whole area; UA, ulce area; IA, improved area.

### Pathological analysis

**Figure 8:**
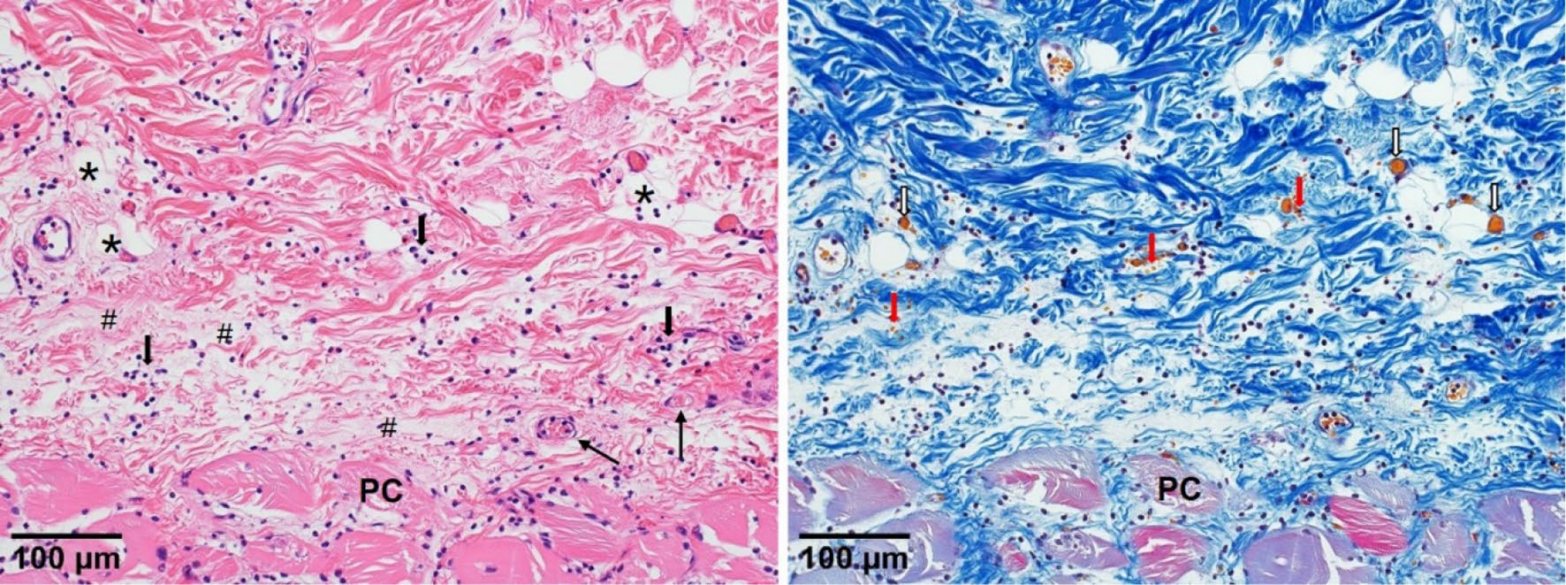
Representative pathological features of early PI. Serial sections were collected and stained with HE (left) and Masson’s trichrome staining (right). Infiltration of polymorphonuclear leukocytes (black arrow) and focal necrosis of the subcutaneous fat (*) and edema (#) were seen in HE staining. In addition, panniculus carnosus muscle (PC) necrosis were also observed. Hemorrhage changes (red arrow) and microthrombi (white arrow) were focally observed in Masson’s trichrome staining.

**Figure 9:**
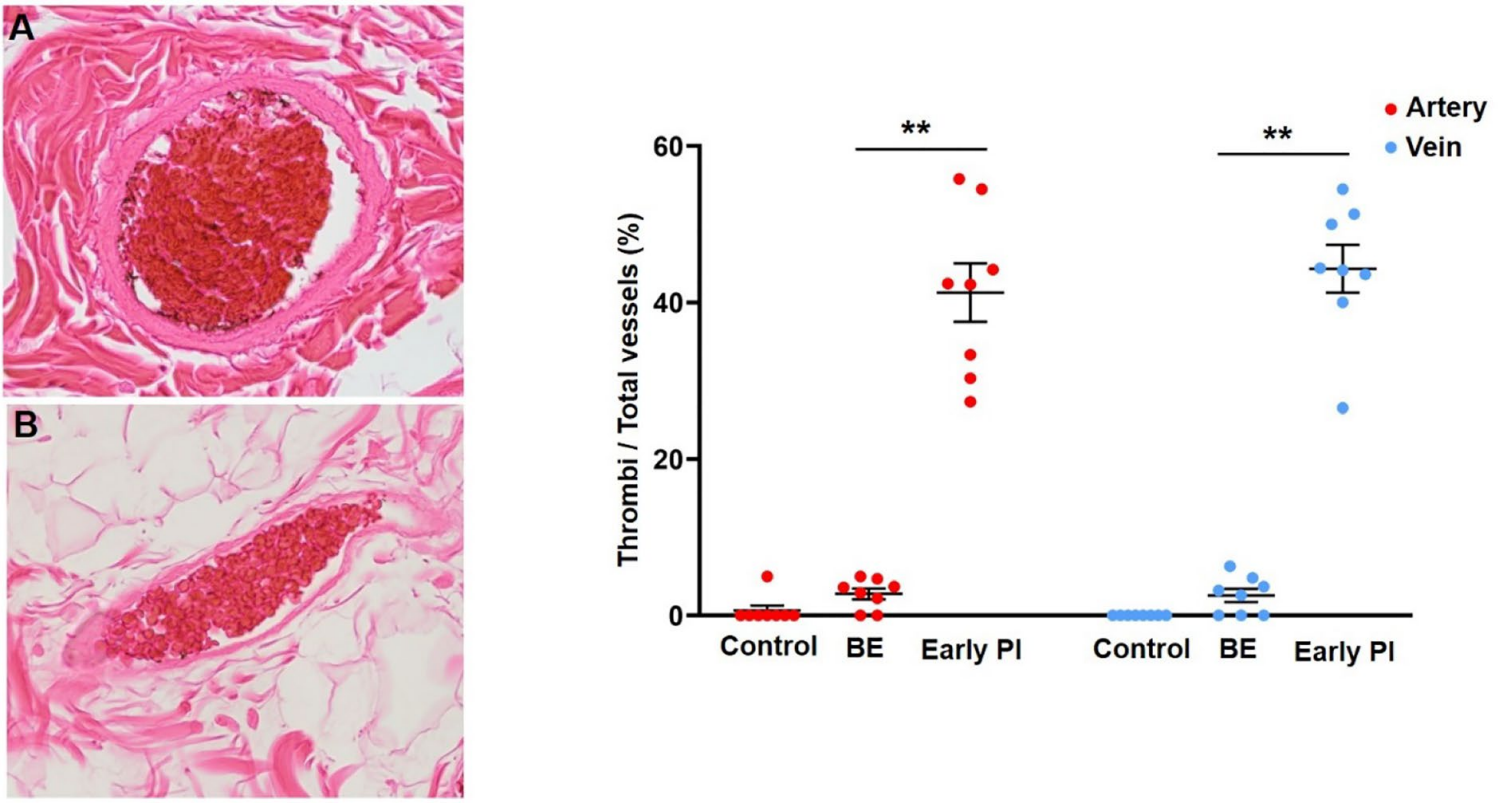
Representative micrographs of pathological observation and quantification of thrombi. BE, blanchable erythema. Thrombus in veins and artery were observed in (**A**) and (**B**) (left). Thrombus was calculated and expressed as % of the total small arteries. n = 8 for each group. **p < 0.001.

To investigate the histological changes, the sections of the lesions harvested from rats were stained with HE. Under a light microscope, HE staining revealed the infiltration of polymorphonuclear leukocytes, focal necrosis of the subcutaneous fat, and extensive necrosis of panniculus carnosus. Small vessels in the dermis became engorged with RBCs with leukocytes stick to the wall. Masson’s trichrome staining showed a diffuse distribution of many orange-red RBCs in the dermis and subcutaneous tissue, as well as a large number of tiny vessels filled with poorly constructed hematocrit components, which we presumed to be microthrombi (Fig. 8). We also noted a large number of small arteriovenous thrombi in the early PI group (*p* < 0.001 vs. BE group), although these were largely absent in the BE group (Fig. 9).

## Discussion

There is still no satisfactory method for detecting multiple circulatory disorders in early PI lesions due to their progression and interaction. We applied UV photography, the CRTT, and the transparent disc method to achieve comprehensive detection of early PI by assessing hyperemia, hemorrhage, congestion, and ischemia. We found that ischemia occurs quickly and persistently after decompression and is valuable for identify early PI before hemorrhage, which is a sign of deterioration.

The comprehensive analysis of the transparent disc method and CRTT not only showed dynamic changes in early PI but also proved to be important tools for differentiation from BE. Within the same erythema, the ulcer area and improved area behave very differently. In addition, we also identified substantial thrombosis formation of arteries, veins, and capillaries in early PI lesions, which may be important factors associated with the involvement of persistent ischemia, congestion, and subsequent hemorrhage after decompression.

According to the guideline of the NPIAP, early PI is defined as intact skin with a localized area of NBE, as measured by the transparent disc method, which is caused by intradermal hemorrhage and can be distinguished from the BE caused by hyperemia^1^. A visual skin assessment has been the standard method for identifying BE and NBE. However, visual skin inspection is difficult and unreliable, as it is likely that severe skin damage has already been sustained before being identified^3,4^. A visual inspection alone is a subjective indicator that is inherently difficult to quantify^5^. There is thus an urgent clinical need to develop accurate assessment methods to improve the ability to identify early PI. However, PI lesions show marked variability. Defloor et al.^10^. had evaluated a photograph of early PI lesions according to the EPUAP classification with 473 nurses and obtained a multirater-Kappa of 0.37, showing very low interrater reliability, with NBE often confused with BE or other lesions. They hypothesized that this inconsistency may be due to changes in the skin or the wound itself rather than inter-observer inconsistency. Our animal experiments also confirmed that early PI, in the course of deterioration to ulceration, and congestion due to reperfusion or inflammation, for example, can be confused for BE, as BE is not completely discolored when pressure is applied, but only the illusion of a color difference between the pressure and non-pressure areas, especially when the color difference is small, and also manifests as NBE immediately after decompression^11^. Such misjudgements can naturally affect the clinical diagnosis, especially as early PI before hemorrhage is complete is more challenging.

Early PI deterioration occurs as a process and reportedly can take up to 48 h from the onset of injury to its visual detection^2^. Our study showed that progression to ulceration also takes more than 36 h. During this process, ischemia–reperfusion injury is considered to be one of the causes of pressure ulcers^1^.The cytotoxic activity of the excess free radicals from restoration blood flow leads to a sterile inflammatory reaction causing the loss of some cell types^12^. In addition, multiple circulatory disorders are involved and interact with each other. As is shown in Fig. 9, recent studies have revealed that, in addition to the above-mentioned hemorrhage and hyperemia, congestion and ischemia are also found within lesions^8,9^. Our previous study showed that congestion appears earlier than hemorrhage after decompression but disappears quickly (probably due to the perfusion of the collateral circulation) and congestion also has its own corresponding impact on the early diagnosis, such as increasing UV shadows when the transparent disc method is used (Fig. 5).

Ischemia after decompression is an important pathology of early PI that should be taken into consideration^9^. However, while ischemia is an important factor associated with the prognosis, its symptoms, such as pale skin masked by more intense erythema and low skin temperature replaced by reperfusion along with an increased skin temperature due to inflammation, are elusive, occasionally causing ischemia to be hidden among other disorders, and there is a distinct and lack of effective detection methods. Ischemia is mainly caused by arterial and capillary thrombosis blocking the blood supply, so stasis of blood due to venous thrombosis may be indirectly involved. Parish et al.^13^ noted that deformation of the skin tissue during negative pressure or shear forces causes excessive stretching and injury to the vascular endothelium, leading to thrombosis. In addition, many studies have reported the presence of thrombosis. Reichel et al.^6^ reported that pressure injury was associated with multiple thrombosis. A pathological specimen examination revealed multiple thrombi in the small vessels of an early PI case^7,9^. Our study also conclusively demonstrated the presence of most arterial but also venous thrombi in early PI lesions (Figs. 8, 9), lesions that are important due to their involvement in the persistent ischemia and exacerbation of subsequent hemorrhage after decompression. Gefen et al.^3,4^ recently showed that secondary inflammation can exacerbate ischemia after decompression. In our study, we found inflammatory cell infiltration and edema, which also contribute to tissue injury and increase tissue pressure, thereby exacerbating ischemia (Fig. 8). Based on MRI findings, Loerakker et al.^14^ suggested that tissue reperfusion after prolonged compression might be incomplete, allowing the ischemia state to continue and thereby exacerbating the injury. Our recent study also verified the presence of severe hypoperfusion in the early PI group after decompression as well as its persistence during deterioration^9^.

Taken together, the above evidence support the presence of ischemia, but the question remains of how to detect it in lesions where multiple circulatory disorders are present. The CRTT is a rapid method for determining peripheral circulatory disorders at the emergency scene^15,16^. Based on this principle, we also utilized spectroscopic instruments to record the CRTT in early PI and demonstrated their utility for detecting localized ischemia^9^. However, as described above, spectroscopy and MRI are expensive to perform, and this high cost hinders their clinical application.

The principle underlying the absorption of UV light by hemoglobin is widely applied in forensic cases to identify intradermal hemorrhage^17^. Our previous study also used UV photograph to observe early PI and found that hemorrhage could be assessed earlier with this approach than with a visual inspection, but pre-hemorrhage changes remained elusive^8^. In the present study, we applied the transparent disc method coupled with the CRTT and combined with UV photography and image processing to perform a comprehensive analysis of erythema, allowing us to accurately identify ischemia in case of early PI before hemorrhage (Fig. 5). This method, combined with the hemorrhage exacerbation shown by the reduction in reflected light as measured by UV gray values (18 h after decompression in Fig. 4), make it possible to accurately evaluate multi-circulatory disorders throughout the process of early PI (Table 1). Furthermore, a comprehensive analysis also highlighted the dynamics of early PI, showing NBE and refilling deficiency (ischemia) at 0.5 h. Of note, it becomes progressively closer to NBE and increase refilling at 6–12 h, which was more pronounced in the ulcer area. This may be due to the congestion of inflammation and the exacerbating effects of hemorrhage, rather than a true improvement in ischemia in cases of erythema. These phenomena may explain the above mentioned inference in the Defloor study^10^, where that confusion of NBE and BE or other lesions was caused by changes in the wound progression itself. In contrast, the BE group in our study was stable in the range of BE and refilling adequate.

We considered the increase in blood component reflux the release of these compression to be the result of increased inflammation and hemorrhage, causing congestion and the RBCs (hemorrhage) scattered throughout the dermis to be compressed out and back in with the gel, as congested and hemorrhage RBCs also spread around the soft tissue when the gel is compressed^18^. Furthermore, even 50% of the UA at 18 h showed blanchable when pressure was applied along with rapid refilling, creating the illusion of non-PI. At this stage, of course, the predominant pathology had changed from ischemia to significant hemorrhage, and the uneven shadow on UV photography at this time helped to compensate for the inadequate pressure diagnosis and CRTT to improve the ability to identify early PI. The CRTT is performed after the transparent disc method added meaningful observations without causing any additional pain to the patient. The concept of ischemia during post-decompression also provides a new idea for the prevention and treatment of pressure ulcers, such as the use of antiplatelet drugs may become a promising potential treatment.

Of note, the Dermo-camera we used in the study is a commercialized dermatologist's tool that was immediately available for clinical use. Although the absence of a function for setting the pressure was a shortcoming.

In addition, the BE which is hyperemia with a small residual UV shadow that should have disappeared on compression seems contradictory (Fig. 5). The same phenomenon is seen in normal skin (data not shown). We hypothesize that this is not a residual blood component in the tissue, but rather a result of the dermis and subcutaneous fat being thinned by pressure, bringing the deeper panniculus carnosus (PC) muscle, which lies outside of the range of UV irradiation, closer to the surface. This is because myoglobin is highly consistent with hemoglobin in terms of its UV light absorption properties^19^. The diagnosis of early PI is also affected by this factor, but NBE is mainly caused by congestion or hemorrhage. The PC muscle, which lies in a relatively deep position, is not exposed to as much UV light as the more residual blood in the compression, therefore, the effect on myoglobin is not significant. There are also vessels that are not squeezed out as much as gels, despite the distortion of the retraction^17^. Thrombosis is thus another factor involved in NBE. The petechial dots and telangiectatic streaks observed by Inui et al.^20^ using dermoscopy, which did not disappear on mild compression, suggest a possible thrombus in a small artery or vein, which is the basis of our inference (Table 2). However, the possibility of UV radiation penetrating the pressure-thinned skin to the deeper tissues, such as the dermatome, also may be relevant to the observation of deep tissue injury (DTI), which will be explored in the future. Furthermore, this study is performed on animals, it still have some difficulties on humans.

**Table 2:** The multi-circulatory disorders of early PI, comparison of observation indexes and mechanism.

The multi-circulatory disorders of early PI, comparison of observation indexes and mechanism								
	Main causes	Occurrence period	Pressure (A/B)	CRTT (B/C)	UV shadow		Outcome	Diagnostic significance
Hyperemia	Reperfusion Inflammation	Early middle ~ late	BE (< 1)	Enough refilling (> 1)	Strong	Uniform shadow	Reversible	±
Ischemia	Arterial thrombus and Microthrobi	Early ~ late	(–)	Refilling delay (< 1)	Pan-white area		Irreversible	+ + +
Congestion	Venous thrombus	Early ~ middle	NBE (> 1)	Refilling delay (< 1 )	Strong	Uneven shadow	Reversible	±
Hemorrhage	Vessel rupture	Middle ~ late (aggravation)	NBE (> 1) ↓ BE (< 1)	Refilling delay (< 1 ) Refilling (> 1)		Uneven shadow	Irreversible	+ + +

**Figure 10:**
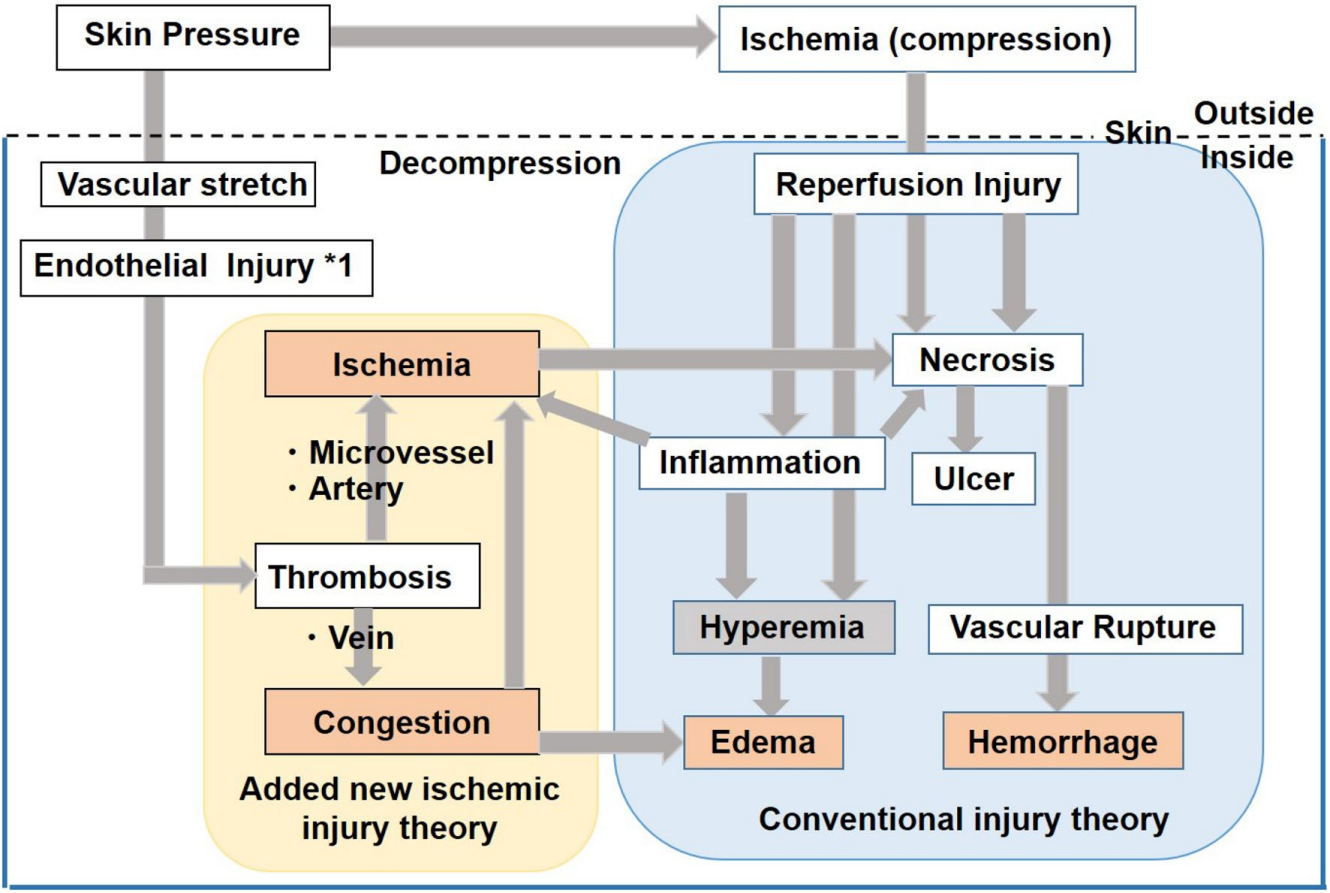
Causes and associations of multi-circulatory disorders in early PI. *1 from Reference^12^.

Finally, it should be stressed that the observation of early PI is moving to the molecular level^12^, which is a huge development. However, a comprehensive analysis of the pathology (Fig. 10), such as circulatory disorders, is still needed in order to achieve a holistic understanding. A more detailed analysis should therefore be conducted to fully grasp the pathological changes in early PI, and the two complement each other can avoid the one-sided analysis.

## Conclusions

In this study, the use of the transparent disc method and CRTT combined with UV photography enabled the comprehensive analysis of complex circulatory disorders during early PI deterioration, and showed that the persistent ischemia post-decompression and subsequent hemorrhage are both important for making a diagnosis, albeit at different times, providing a new approach to and scientific basis for the clinical diagnosis and prediction of the prognosis.

The structure of animal skin is different from that of humans. Therefore, this method still needs to be validated in a clinical setting. At the same time, the Dermo-camera is not able to set pressure, an issue that needs to be addressed in the further. This device is also quite expensive, so a cheaper device needs to be developed for widespread clinical use.

### Supplementary Information

Supplementary Table 1.

## Data Availability

The data that support the findings of this study are available on request from the corresponding author. The data are not publicly available due to privacy or ethical restrictions.
