# Supplementary material for: A comprehensive analysis of multi-circulatory disorders in early pressure injury and their diagnostic significance in rat models
Source: Sci Rep. 2023 Nov 7;13:19342. doi: 10.1038/s41598-023-46676-x (PMC10630315; doi:10.1038/s41598-023-46676-x)
Supplement: Supplementary file 1 — Supplementary Table 1. [file 41598_2023_46676_MOESM1_ESM.docx]

|  |  |  |  |  |  |  |  |  |  |  |  |  |  |  |
| --- | --- | --- | --- | --- | --- | --- | --- | --- | --- | --- | --- | --- | --- | --- |
|  | | | After decompression | | | | | | | | | | | |
|  |  |  | 0 min | | | 5 min | | | 10 min | | |  | | |
|  |  |  | Pre-pressure | Pressure load | Post-depression | Pre-pressure | Pressure load | Post-depression | Pre-pressure | Pressure load | Post-depression |  |  |  |
| Gray value | BE | WA | 162.3±1.7 | 167.8±2.4 | 154.6±2.3 | 174±1.7 | 181.9±1.5 | 164.6±2.5 | 189.0±2.2 | 190.2±1.7 | 176.9±2.3 |  |  |  |
|  | PI | WA | 168.6±2.6 | 153.9±2.0 | 159.1±1.8 | 174.3±3.4 | 166.9±2.9 | 165.6±2.8 | 166.2±3.0 | 163.7±2.9 | 160.7±3.0 | 164.7±2.5 | 163.7±2.6 | 153±2.2 |
|  |  | UA | 160.9±2.7 | 142.2±1.8 | 152.8±2.2 | 171.4±3.0 | 159.8±2.8 | 159.8±2.6 | 162.9±2.6 | 155.9±3.2 | 158.6±2.9 | 152±3.5 | 148.2±4.0 | 142.8±3.5 |
|  |  | IA | 170.1±2.6 | 156.5±2.1 | 160.8±1.6 | 175±3.5 | 168.4±2.9 | 167.1±2.8 | 167.1±3.1 | 165.4±2.8 | 161.3±3.1 | 168±2.7 | 167.4±2.2 | 155.4±2.2 |
|  | | | Pre-pressure | Pressure load | Post-depression | Pre-pressure | Pressure load | Post-depression | Pre-pressure | Pressure load | Post-depression | Pre-pressure | Pressure load | Post-depression |
|  |  |  | 0.5 h | | | 6 h | | | 12 h | | | 18 h | | |
|  |  |  | After decompression | | | | | | | | | | | |

Supplementary table 1. Specific data of UV gray values by the transparent disc method and CRTT. Gray values at different times after decompression with transparent disc method and CRTT in the two groups. The UV gray value represents three points: pre-pressure, pressure load (150 mmHg) and after depression. In BE group, the results showed the gray values at the start, 5 min and 10 min after decompression using CRTT. In the early PI group, the results showed the gray values of WA, UA and IA at different times after decompression. WA: whole area; UA, ulcer area; IA, improvement area. n = 10 for each group. BE: blanchable erythema.
